# Motion and Inertia Estimation for Non-Cooperative Space Objects During Long-Term Occlusion Based on UKF-GP

**DOI:** 10.3390/s25030647

**Published:** 2025-01-22

**Authors:** Rabiul Hasan Kabir, Xiaoli Bai

**Affiliations:** Department of Mechanical and Aerospace Engineering, Rutgers University, Piscataway, NJ 08854, USA; rabiulhasan.kabir@rutgers.edu

**Keywords:** Gaussian process, unscented Kalman filter, proximity operation, non-cooperative tumbling space object, space-domain awareness

## Abstract

This study addresses the motion and inertia parameter estimation problem of a torque-free, tumbling, non-cooperative space object (target) under long-term occlusions. To solve this problem, we employ a data-driven Gaussian process (GP) to simulate sensor measurements. In particular, we implement the multi-output GP to predict the projection measurements of a stereo-camera system onboard a chaser spacecraft. A product kernel, consisting of two periodic kernels, is used in the GP models to capture the periodic trends from non-periodic projection data. The initial guesses for the periodicity hyper-parameters of the GP models are intelligently derived from fast Fourier transform (FFT) analysis of the projection data. Additionally, we propose an unscented Kalman filter–Gaussian process (UKF-GP) fusion algorithm for target motion and inertia parameter estimation. The predicted projections from the GP models and their derivatives are used as the pseudo-measurements for UKF-GP during long-term occlusion. Results from Monte Carlo (MC) simulations demonstrate that, for varying tumbling frequencies, the UKF-GP can accurately estimate the target’s motion variables over hundreds of seconds, a capability the conventional UKF algorithm lacks.

## 1. Introduction

In recent years, developing autonomous strategies for proximity operations between two spacecraft, such as docking, refueling, repairing, or robotic capture of space debris, has gained tremendous interest due to economic incentives, societal needs, and technological advancement. Such operations typically require physical interactions between two spacecraft. Consequently, it is important to have good knowledge of the motion parameters of the spacecraft to create a safe policy for the approach and interaction segments of the operation. Proximity operations between two cooperative spacecraft can be executed relatively easily due to both spacecraft being able to relay information regarding their motions. However, in proximity operations like space debris removal, the non-cooperative target space object cannot relay any information. It is essential to understand the target’s motion parameters to design safe policies for approach and interaction. Additionally, the target’s physical properties may be unknown and require determination.

Sensors such as the laser camera system (LCS) and mono and stereo-vision camera systems can be utilized to collect data regarding the target. The LCS sensor is less prone to sensor occlusion and has a higher measurement sampling frequency [1]. However, vision-based camera systems are more energy-efficient. This study is conducted with the assumption that the chaser carries a stereo-vision camera system. The stereo images captured by the cameras can be processed through computer vision algorithms, which return information regarding the features of the target. In general, features can either be regions [2], curves or lines [3], or points [4], which have higher visibility than their surrounding areas and, thus, are easily distinguishable in the captured images. In the context of the considered satellite proximity operations, the features indicate salient points located on the surface of the target, such as corners of solar panels and endpoints of the poles on antennas.

To obtain reliable estimates of the motion and physical parameters of a target space object from the sensor measurements, researchers have primarily relied on physics-based methods, which utilize the underlying dynamics equations associated with the target’s motion. Two of the widely utilized algorithms are the extended Kalman filter (EKF) and the unscented Kalman filter (UKF), where the latter does not require the linearization of the non-linear dynamics and observation models. Segal et al. conducted one of the earliest investigations on the estimation of the relative rotational and translational motion of a target object using the iterated EKF (IEKF) using the measurements of a fixed number of point features from a stereo-camera system [5,6]. Multiple IEKF models are run in parallel with a different choice for the inertia parameters, and the best inertia tensor was chosen from a maximum a posteriori (MAP) estimation. Later, Pesce et al. proposed an IEKF-based algorithm where the inertia parameter ratios are estimated without the MAP estimation [7]. They also utilized the optical flow or the rate of projection as measurements, which reduces the number of point features to five for the IEKF model to converge. A similar approach is investigated by Chang et al. in [8], except they considered a varying number of point features. Ge et al. showed in [9] that it is possible to achieve convergence of an EFK model to estimate the target motion from the stereo-camera measurements of only three-point features. Feng et al. developed an EFK model to estimate target motion where two dependent variables parameterize the inertia ratios that fully encode the physical constraints between the inertia parameters [10]. Li et al. proposed an interacting multiple model unscented Kalman filter (IMM-UKF) to estimate the relative motion of a target to deal with uncertainties caused by unknown noise [11]. Mazzucato et al. validated an EKF model for estimating the motion of a target both numerically and experimentally in [12], whereas De Jongh et al. experimentally validated the estimation capability of a SLAM-based EKF algorithm tasked with estimating the relative motion, shape, and size of a target object [13]. Jiang et al. developed a constrained EFK algorithm for estimating the relative motion of a target that uses physical constraints among point features of the target to ensure that the system is observable [14]. A similar geometric constraint among point features was considered by Jixiu et al. for an EKF algorithm to estimate a target object’s relative motion [15]. In [16], Maestrini et al. proposed an algorithm called the coarse model-based relative navigation (CoMBiNa) to create a 3D model and estimate the motion as well as inertia parameters of an unknown and non-cooperative target object. A coarse point-cloud model of the object using ORB-SLAM from stereo-camera measurements was later used as a reference for the UKF model to estimate the rotational and translational motion and the inertia parameters of the target. A similar problem was also addressed in [17] with the consideration of the unscented Kalman filter-based technique and the stereo-camera sensor. Apart from Kalman filter-related techniques, several other physics model-based approaches have been studied for estimating the target motion and inertia parameters, which include the non-linear least square technique [18], the smoothing and mapping factor-graph-based method [19], the constrained convex optimization method [20], etc.

Physics model-based approaches provide a way to estimate motion and physical parameters that are consistent with the underlying physics models. However, these approaches come with some major drawbacks. One of the key issues is the inability of these algorithms to provide reliable estimates of the target states if the sensor measurements are not available for a long time (i.e., hundreds or thousands of seconds). In space, long-term occlusions can happen for numerous reasons, especially for visual sensors. Examples include when the target is between the sun and the chaser or when the target is in the Earth’s shadow while orbiting the Earth. In both of these scenarios, the chaser is unable to observe the target using a visual camera sensor.

With the advancements in machine learning (ML) techniques and computational capabilities, researchers have started investigating the capability of model-free ML techniques. In recent work, Yu et al. implemented the sparse pseudo-input Gaussian process (SPGP) technique to predict the inertial position of a point of interest and a body frame attitude quaternion of a tumbling space object for ten seconds [21]. This method uses a simple RBF kernel, which is not capable of capturing the important trends of the training datasets and, consequently, is not appropriate for long-term occlusion. Researchers have also investigated combining ML and physics model-based approaches to improve parameter estimation performance. An example is the development of a dual-vector quaternion-based mixed artificial neural network (DVQ-MANN) estimation algorithm to assist a dual-vector quaternion-based extended Kalman filter (DVQ-EKF) during sensor failure [22]. The authors demonstrated that this approach helped prevent the associated error of the state variables of the DVQ-EKF from diverging. However, the presented results do not demonstrate if the algorithm is capable of further reducing the estimation errors during occlusions. Additionally, Barbier combined three Gaussian process (GP) models with a two-stage cascading EKF system to estimate the relative motion and create a map of a target object in [23]. After executing the first EKF model, which estimated the relative motion of the chaser, GP models were generated to predict the angular velocity of the target to propagate the second EKF model. However, this study did not consider any occlusion and, hence, did not demonstrate how the estimation algorithm may perform in the absence of sensor measurement.

One of the goals of this study is to predict the projection measurements of a stereo-camera system during occlusion with the help of an ML algorithm, and we select the Gaussian process (GP) for this task. The GP is a non-parametric algorithm defined by a mean function and a covariance or kernel function. For this study, we are interested in an advanced GP regression algorithm called the multi-output GP. The traditional single-output GP model can only learn the correlations among the inputs, but the multi-output GP can capture the correlations among the inputs and the correlations among the outputs. Due to this capability, the multi-output GP model can provide more accurate predictions of a vector-valued function with correlated components from smaller training data sets than single-output GP models. However, the multi-output GP models require longer training time due to the additional task of finding the covariances among the outputs. The projections of the point features on the stereo-camera image planes are functions of the correlated components of the feature positions relative to the cameras, which makes the projection components correlated as well. Therefore, the multi-output GP model is chosen as the data-driven method to predict the projections in this study. On the other hand, GP has been shown to be more accurate at providing predictions compared to neural networks (NNs) if trained from a training dataset of the same size [24], which is another reason for favoring GP in our work.

The primary contribution of this study is the development of a UKF-GP fusion algorithm to estimate the rotational motion variables and the inertia parameters of a non-cooperative, tumbling target space object under long-term occlusion. The following contributions are made in the development of the UKF-GP algorithm:

- We utilize multi-output GP models to predict projection measurements from a stereo-camera system so that the UKF-GP model can be updated during occlusion. GP models are trained using the projection measurements provided by stereo-camera systems. A product kernel, consisting of two periodic kernels, is designed to capture periodic trends in the non-periodic and noisy training data.
- The initial guesses for the periodicity hyper-parameters are intelligently obtained from the FFT analysis of the training data, enhancing the hyper-parameter training procedure.
- The projection predictions from the GP models and their derivatives obtained from the finite difference method (FDM) are used as pseudo-measurements in the UKF-GP fusion algorithm during long-term occlusion.
- Monte Carlo simulations across multiple tumbling frequencies demonstrate that GP models accurately predict projections for thousands of seconds under occlusion. Furthermore, the UKF-GP algorithm outperforms the conventional UFK in estimating motion and inertia parameters.

Note that an algorithm called the GP-UKF is proposed in [25] that also combines UKF and GP to improve the estimation accuracy. In that work, the GP models are used to replace the propagation and observation models of the UKF model. Differently, we use GPs to predict the stereo-camera measurements in this work. Additionally, we remark that the predictions from the GP models can be used as pseudo-measurements for any suitable physics-based estimation algorithm.

The paper is structured as follows: in Section 2, we provide descriptions of the problem scenario, dynamics and observation models, a summary of the UKF algorithm, multi-output GP models, overall UKF-GP frameworks, and the simulation workflow. In Section 3, we present the results from MC simulations to demonstrate the effectiveness of GP models and the UKF-GP algorithm. Finally, in Section 4, we provide our concluding remarks.

## 2. Methodology

In this section, we provide the proposed UKF-GP framework in detail. First, we present the problem scenario, followed by descriptions of the dynamics and observation models for the UKF algorithm. Then, we present a procedure to create GP models that are used for predicting projection measurements. Finally, we discuss the overall UKF-GP framework and the considered simulation workflow for this study.

### 2.1. Problem Scenario

**Figure 1:**
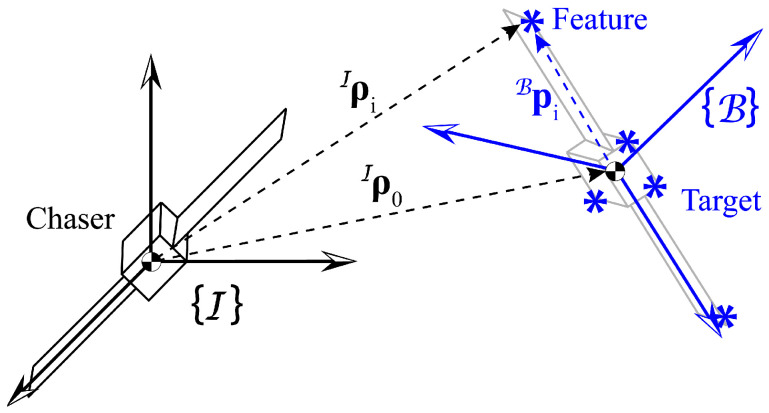
Schematic of the simulation scenario. The left spacecraft (chaser) is a cooperative spacecraft carrying a stereo-camera system, and the right one (target) is a torque-free, tumbling, non-cooperative space object. I is the inertial frame, and B is the body frame of the target that is parallel to the principal axes of the target. The target’s point features are indicated by the asterisk symbols.

In this paper, we aim to estimate the relative rotational motion of a target space object with respect to a chaser spacecraft. Hence, we assume that both the chaser and the target center of mass (CM) are inertially fixed, and the relative position of the target CM with respect to the chaser CM is exactly known. Also, the chaser is presumed to be rotationally fixed. The overall scenario is illustrated in Figure 1. An inertial frame I is defined with the origin being located at the chaser CM. Additionally, the B frame is the principal axe’s body frame of the target, which has the origin at the target CM. The attitude quaternion of B with respect to I is $q={\left[q_{s},q_{x},q_{y},q_{z}\right]}^{T}$. The corresponding rotation matrix of q is defined as $C\left(q\right)=C_{I}^{B}$, such that νB=CIB·νI, where νI and νB indicate an arbitrary vector expressed in I and B. The angular velocity associated with $\dot{q}$ is denoted by ωI and ωB in I and B, respectively. The relative position of the target CM with respect to the chaser CM in I is ρ0I=[x0,y0,z0]T, which is known, as mentioned earlier. The position of the *i*-th point feature with respect to the target CM in B and the chaser CM in I are defined as piB and ρiI. The relationship between piB and ρiI is given as follows:

(1) ρiI=ρ0I+[CIB]T·Bpi.

### 2.2. Unscented Kalman Filter (UKF)

Since we do not aim to modify the conventional UKF algorithm in this paper, we present the dynamics and observation models of the UKF model in this section while skipping the overall UKF algorithm. There exist numerous books and papers that discuss the theoretical derivation and the practical implementation of the UKF algorithm, such as [26,27], which the reader can consult to get an in-depth understanding of the UKF algorithm.

#### 2.2.1. Dynamics Model

This study focuses on estimating the relative rotational motion and inertia parameters of the target space object; hence, the state variable X is defined as follows:

(2) X=[ωBT,qT,k1,k2,{piTB}i=1nf]T,

where $n_{f}$ is a constant that indicates the number of features. Also, $k_{1}$ and $k_{2}$ are the inertia parameter ratios, as defined in [19]. Tweddle [19] showed that it is not possible to observe all three principal axes of inertia parameters of a tumbling object without applying external torque, and only two out of three degrees of freedom are observable. Hence, they introduced two inertia parameter ratios, which are defined as follows: given that $I_{1}$, $I_{2}$, and $I_{3}$ are the principal axes inertia parameters and the inertia matrix I in B is

(3) $$I=\left[\begin{array}{ccc} I_{1} & 0 & 0\\ 0 & I_{2} & 0\\ 0 & 0 & I_{3} \end{array}\right],$$

then

(4) $$k_{1}=\log\left(\frac{I_{1}}{I_{2}}\right),\;k_{2}=\log\left(\frac{I_{2}}{I_{3}}\right).$$

Accordingly, I can be rewritten in terms of $k_{1}$ and $k_{2}$ as follows:

(5) $$I=\left[\begin{array}{ccc} I_{1}/I_{2} & 0 & 0\\ 0 & 1 & 0\\ 0 & 0 & I_{3}/I_{2} \end{array}\right]=\left[\begin{array}{ccc} \exp(k_{1}) & 0 & 0\\ 0 & 1 & 0\\ 0 & 0 & \exp(-k_{2}) \end{array}\right].$$

The torque-free rotational dynamics equations describing the rate of change of ωB are given as follows:

(6) ω˙B=I−1([ωB⊗]I·ωB)=exp(−k1)(1−exp(−k2))ωyBωzB(exp(−k2)−exp(k1))ωxBωzBexp(k2)(exp(−k1)−1)ωxBωyB,

where

(7) [ωB⊗]=0−ωzBωyBωzB0−ωxB−ωyBωxB0,

with ωxB, ωyB, and ωzB being the components of ωB. Note that this work does not consider perturbations such as the gravity of the Earth or atmospheric drag.

The rotational kinematics equation describes the rate of change in q, which is mathematically written as follows:

(8) $$\dot{q}=\frac{1}{2}\Omega q,$$

where

(9) Ω=0−ωxB−ωyB−ωzBωxB0ωzB−ωyBωyB−ωzB0ωxBωzBωyB−ωxB0.

We assume that the target object is a rigid body; therefore, we can ignore the movements of the internal components. Under this assumption, the rates of change in the inertia parameter ratios and the feature positions in B are given as follows:

(10) $$\begin{array}{cc} {\dot{k}}_{1} & =0, \end{array}$$

(11) $$\begin{array}{cc} {\dot{k}}_{2} & =0, \end{array}$$

(12) p˙iB=03×1.

For the UKF model, we consider a discrete-time dynamics model for propagating state variables. Mathematically, the state variable X(k+1) at time-step k+1 is written as follows:

(13) $$X\left(k+1\right)=X\left(k\right)+\int_{t=t(k)}^{t=t(k)+\Delta t}f\left(X\left(k\right)\right)dt+w\left(k\right),$$

where Δt is the discrete time-interval, and t(k) and w(k) are the time instance and the zero mean Gaussian white noise denoting the process noise at time step *k*, respectively. Additionally, f(X) is obtained from Equations (6)–(12) and written as follows:

(14) f(X)=I−1([ωB⊗]I·ωB)12Ωq0(3nf+2)×1.

The process noise covariance matrix Q is defined as a constant diagonal matrix and written as follows:

(15) $$Q=E\left[ww^{T}\right]=diag\left(\left[\Sigma_{\omega }^{Q},\Sigma_{q}^{Q},\Sigma_{k_{1}}^{Q},\Sigma_{k_{2}}^{Q},\Sigma_{p_{1}}^{Q},\ldots ,\Sigma_{p_{n_{f}}}^{Q}\right]\right),$$

where $\Sigma_{\omega }^{Q}\in R^{3}$, $\Sigma_{q}^{Q}\in R^{4}$, $\Sigma_{k_{1}}^{Q}\in R$, $\Sigma_{k_{2}}^{Q}\in R$, and ${\left\{\Sigma_{p_{i}}^{Q}\in R^{3}\right\}}_{i=1}^{n_{f}}$ contain the process noise variances of ωB, q, $k_{1}$, $k_{2}$, and {piB}i=1nf. Note that the initial state error covariance matrix $P_{0}$ has the same size as Q, and the vectors containing the corresponding error variances of $P_{0}$ are denoted by the same symbols as those used for Q, except the superscript ‘*Q*’ is replaced by the superscript ‘*P*’.

#### 2.2.2. Observation Model

In this study, we assume that the chaser is equipped with a stereo-vision camera system to collect information regarding the chaser. The stereo images captured by the stereo-camera system can be processed by computer vision algorithms to detect point features and perform feature matching. There exist a number of methods for feature detection, such as SIFT [28], SURF, AKAZE, ORB [29], etc. In this work, we do not implement feature detection methods and assume that the noisy projection data of the features are available from image processing algorithms.

**Figure 2:**
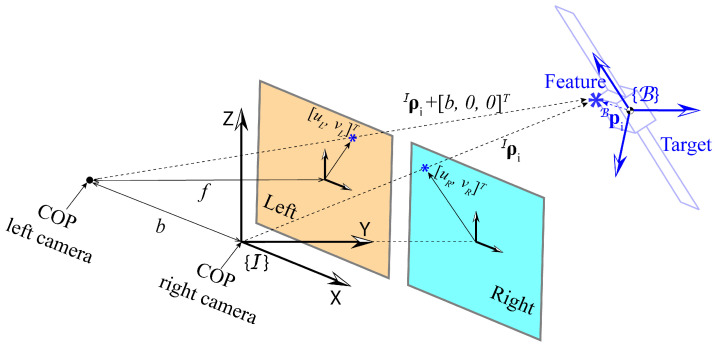
Schematic of the stereo-vision camera system.

The detected feature positions are obtained as projections on camera planes. The schematic showing the onboard stereo-camera system is illustrated in Figure 2. The working mechanism of the cameras is based on the pinhole camera model. The center of projection (COP) of the right camera is assumed to be located at the chaser CM or the origin of I. Additionally, we assume that the camera frame C is aligned with I at any time since the chaser does not perform any rotation. The COP of the left camera is located at ${\left[-b,0,0\right]}^{T}$, expressed in I. The image planes are located perpendicular to the *Y* axis with a focal length *f* distance from the corresponding COPs. Let the *i*-th feature located at piB in B have the position vector ρiI=[xi,yi,zi]T at time-step *k*. Then, the projection of the feature on the right (left) camera plane is defined as the intersecting point of ρiI (ρiI+[b,0,0]T) and the right (left) camera plane. The 2D projection vectors of the feature on the image planes are denoted by $w_{R,i}={\left[u_{R,i},v_{R,i}\right]}^{T}$ and $w_{L,i}={\left[u_{L,i},v_{L,i}\right]}^{T}$ for the right and the left cameras, respectively, where $u_{\left(\cdot \right)}$ and $v_{\left(\cdot \right)}$ indicate the projection components in the horizontal and vertical directions on the image planes. The expressions of $u_{R,i}$, $v_{R,i}$, $u_{L,i}$, and $v_{L,i}$ are given as follows:

(16) $$\begin{array}{cc} u_{R,i} & =f\frac{x_{i}}{y_{i}},\;v_{R,i}=f\frac{z_{i}}{y_{i}}, \end{array}$$

(17) $$\begin{array}{cc} u_{L,i} & =f\frac{x_{i}+b}{y_{i}},\;v_{L,i}=f\frac{z_{i}}{y_{i}}. \end{array}$$

In practice, one can numerically calculate the optical flows or the rates of change in the projections, ${\dot{w}}_{R,i}$ and ${\dot{w}}_{L,i}$ from $w_{R,i}$ and $w_{L,i}$ (e.g., using the finite difference method). Therefore, the optical flows ${\dot{w}}_{R,i}$ and ${\dot{w}}_{L,i}$ are included in the observation model in this study similar to [7,30]. The expressions of ${\dot{w}}_{R,i}$ and ${\dot{w}}_{L,i}$ are derived in a similar way to that shown in [31], which are given below:

(18) w˙R,i=u˙R,iv˙R,i=AR,i·ωI,

(19) w˙L,i=u˙L,iv˙L,i=AL,i·ωI,

where

(20) $$\begin{array}{cc} A_{R,i} & =\left[\begin{array}{ccc} \frac{x_{i}\left(z_{i}-z_{0}\right)}{y_{i}^{2}} & \frac{\left(z_{i}-z_{0}\right)}{y_{i}} & -\left(\frac{y_{i}-y_{0}}{y_{i}}+\frac{x_{i}\left(x_{i}-x_{0}\right)}{y_{i}^{2}}\right)\\ \left(\frac{y_{i}-y_{0}}{y_{i}}+\frac{z_{i}\left(z_{i}-z_{0}\right)}{y_{i}^{2}}\right) & -\frac{\left(x_{i}-x_{0}\right)}{y_{i}} & -\frac{z_{i}\left(x_{i}-x_{0}\right)}{y_{i}^{2}} \end{array}\right], \end{array}$$

(21) $$\begin{array}{cc} A_{L,i} & =\left[\begin{array}{ccc} \frac{\left(x_{i}+b\right)\left(z_{i}-z_{0}\right)}{y_{i}^{2}} & \frac{\left(z_{i}-z_{0}\right)}{y_{i}} & -\left(\frac{y_{i}-y_{0}}{y_{i}}+\frac{\left(x_{i}+b\right)\left(x_{i}-x_{0}\right)}{y_{i}^{2}}\right)\\ \left(\frac{y_{i}-y_{0}}{y_{i}}+\frac{z_{i}\left(z_{i}-z_{0}\right)}{y_{i}^{2}}\right) & -\frac{\left(x_{i}-x_{0}\right)}{y_{i}} & -\frac{z_{i}\left(x_{i}-x_{0}\right)}{y_{i}^{2}} \end{array}\right]. \end{array}$$

The complete derivation process of Equations (18)–(21) is provided in Appendix A.

Together, Equations (16)–(19) for $n_{f}$ number of features define the observation model h(X):

(22) $$h\left(X\left(k\right)\right)={\left[h_{1}^{T},h_{2}^{T},\ldots ,h_{n_{f}}^{T}\right]}^{T},$$

where, for any $i=1,2,\ldots ,n_{f}$,

(23) $$h_{i}={\left[w_{R,i}^{T},\;w_{L,i}^{T},\;\left(u_{L,i}-u_{R,i}\right),\;{\dot{w}}_{R,i}^{T},\;{\dot{w}}_{L,i}^{T}\right]}^{T}.$$

The measurement model is defined as follows:

(24) z(k)=h(X(k))+v(k),

where $v\left(k\right)\in R^{9n_{f}}$ is the measurement noise with components being sampled from Gaussian distributions with zero mean and standard deviations $\sigma_{p}$, $\sigma_{d}$, and $\sigma_{o}$ for projections, disparity, and optical flows. The measurement noise covariance matrix is written as follows:

(25) $$R=E[vv^{T}],$$

where R is a diagonal matrix, which is expressed in terms of $\sigma_{p}$, $\sigma_{d}$, and $\sigma_{o}$.

### 2.3. The Gaussian Process (GP)

The GP is a Bayesian regression technique that is traditionally implemented to generate a predictive distribution of a scalar function over the single or multi-dimensional input domain. In other words, a GP model returns a scalar output’s mean and uncertainty at a given input. In achieving this, a covariance function or kernel is required to determine the correlations among the inputs in the training data. In a broader sense, a kernel helps capture useful features such as smoothness, periodicity, etc., regarding the underlying output function from the discrete training data. Hence, selecting an appropriate kernel to create a GP model with good prediction ability is crucial.

Multiple single-output GP models can be generated to predict a vector-valued function where each GP model is tasked with estimating one function component. This approach is ineffective if the components of the function are correlated due to the single-output GP models’ inability to capture the correlation among the output domains, thus requiring relatively bigger training data sets. To create prediction models of a vector-valued function with correlated components, it is more data-efficient to implement an advanced version of GP called the multi-output GP, which depends on an additional kernel that detects the correlations among the outputs. However, training a multi-output GP model takes longer due to the additional covariance determination task for the output dimensions.

In this work, we aim to predict the right and left camera projections of the features, ${\left\{w_{R,i}\right\}}_{i=1}^{n_{f}}$ and ${\left\{w_{L,i}\right\}}_{i=1}^{n_{f}}$. Intuitively, the components of $w_{R,i}$ and $w_{L,i}$ are correlated since they are functions of piI whose components are correlated as well. Therefore, it is advantageous to generate a multi-output GP model $G_{i}$ for the right and left projections of each feature, which can find the correlations among the components of the projection measurements. For simplification, let us define a vector $w_{i}={\left[u_{R,i},v_{R,i},u_{L,i}\right]}^{T}$ for the *i*-th feature so that all projection components are included in one vector. We do not include $v_{L,i}$ in $w_{i}$ because $v_{L,i}=v_{R,i}$ by definition.

To train the GP model $G_{i}$ for the *i*-th feature, a dataset $D_{i}$ is required, which contains the projection measurements ${\tilde{w}}_{i}$. The dataset $D_{i}$ is four-dimensional with time as the input and three projection components of ${\tilde{w}}_{i}$ as the output. The format of the dataset $D_{i}$ is shown as an example in Table 1.

We use the intrinsic coregionalization model (ICM) kernel [32] to determine the covariance among output domains. Also, we utilize a product kernel as the covariance function for the input domain for each of the GP models. The kernel is formulated as the product of two periodic kernels. The product kernel $K(t_{i},t_{j})$ for $G_{i}$ is given as follows:

(26) $$K\left(t_{i},t_{j}\right)=\sigma_{1}^{2}\exp\left(-\frac{2}{l_{1}^{2}}\sin^{2}\left(\frac{\pi \delta t}{P_{1}}\right)\right)\cdot \sigma_{2}^{2}\exp\left(-\frac{2}{l_{2}^{2}}\sin^{2}\left(\frac{\pi \delta t}{P_{2}}\right)\right),$$

where $\delta t=|t_{i}-t_{j}|$. Also, $l_{1}$ and $l_{2}$ are the length-scale hyper-parameters, $\sigma_{1}$ and $\sigma_{2}$ are the smoothness hyper-parameters, and $P_{1}$ and $P_{2}$ are the periodicity hyper-parameters. The hyper-parameters are obtained from the maximum likelihood optimization operation performed on the training dataset [33].

Note that, for a torque-free tumbling object, the components of the position of an arbitrary point located on the object expressed in an inertial frame are not periodic functions [34]. Hence, the components of ρiI are non-periodic. However, the underlying reason behind the variations of ρiI components is the angular velocity of the target, which is periodic if expressed in B, with the periodicity being the polhode period $T_{p}$. Hence, we speculate that even though the components of ρiI are non-periodic, they should have some intrinsic periodic properties, which we want to capture using the periodic kernels. The logic behind using two periodic kernels in the product kernel is that one periodic kernel should capture the effect of the tumbling rate or the magnitude of the angular velocity while the other periodic kernel should learn the effect of the polhode period on the variations of $w_{i}$.

Although the periodicity hyper-parameters $P_{1}$ and $P_{2}$ in Equation (26) can be determined by hyper-parameters training from the given training datasets, it significantly improves the performance of the trained GP models if we provide good initial guesses of the periodicity hyper-parameters. This reduces the chance of the hyper-parameter training optimization algorithm getting stuck at local solutions. Hence, we utilize a fast Fourier transform-based strategy to determine the initial guesses of $P_{1}$ and $P_{2}$. The formal approach to obtain the initial guesses of $P_{1}$ and $P_{2}$ for the product kernel *K* is provided in Algorithm 1.
**Algorithm 1** Periodicity hyper-parameters initial guess determination algorithm for ρiI.  1:**for** j=1to3  **do**  2:      Perform the fast Fourier transform (FFT) operation on the *j*-th component of ${\tilde{w}}_{i}={\left[{\tilde{u}}_{R,i},{\tilde{v}}_{R,i},{\tilde{u}}_{L,i}\right]}^{T}$  3:      Determine the frequencies $f_{j1}$ and $f_{j2}$ with the highest and second highest magnitudes from the FFT operation  4:      Calculate the corresponding periods $P_{j,1}$ and $P_{j,2}$ of $f_{j,1}$ and $f_{j,2}$ as $P_{j,(\cdot )}=1/f_{j,(\cdot )}$  5:**end for**  6:Obtain the unique periods from $\left[{\left\{P_{j1}\right\}}_{j=1}^{3},{\left\{P_{j2}\right\}}_{j=1}^{3}\right]$  7:Create all possible combinations of a pair of periods from the list of unique periods.  8:Calculate the absolute difference between the periods and the sum of magnitudes provided by FFT for each pair of unique periods.  9:Multiply the absolute time difference and the sum of the magnitudes10:Select the pair of unique periods with the highest product as the initial guesses of the periodicity hyper-parameters.

### 2.4. UKF-GP Algorithm

**Figure 3:**
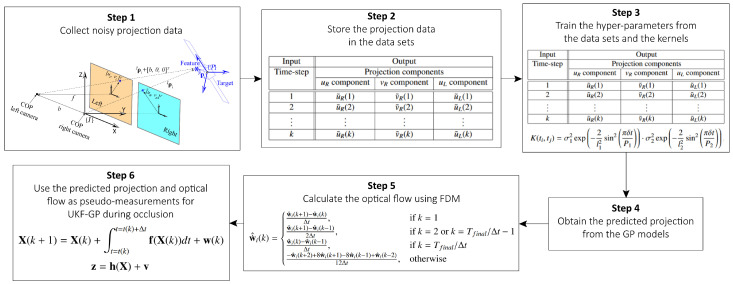
Flowchart of the UKF-GP algorithm.

The flowchart of the proposed UKF-GP algorithm is presented in Figure 3. In the first step, the noisy projection data ${\left\{{\tilde{w}}_{i}\left(k\right)\right\}}_{i=1}^{n_{f}}$ are collected from time step k=1 (equivalently, t=0) to $k=T_{train}/\Delta t$ or $t=T_{train}$ with the measurement frequency $f_{s}=1/\Delta t$. Then, the collected noisy projection measurements are stored in the dataset ${\left\{D_{i}\right\}}_{i=1}^{n_{f}}$. Next, the hyper-parameters of the GP models ${\left\{G_{i}\right\}}_{i=1}^{n_{f}}$ are trained from the datasets ${\left\{D_{i}\right\}}_{i=1}^{n_{f}}$ and the kernel functions. In the next step, the predictions of the projections ${\left\{{\hat{w}}_{i}\left(k\right)\right\}}_{i=1}^{n_{f}}$ are obtained from ${\left\{G_{i}\right\}}_{i=1}^{n_{f}}$. The predictions of the optical flows ${\left\{{\hat{\dot{w}}}_{i}\left(k\right)\right\}}_{i=1}^{n_{f}}$ are then calculated from ${\left\{{\hat{w}}_{i}\left(k\right)\right\}}_{i=1}^{n_{f}}$ using the finite difference method (FDM). We implement the first-order forward and backward differences as well as the second and fourth-order central differences [35] to compute the optical flows, and the corresponding FDM equations are given as follows:

(27) $${\hat{\dot{w}}}_{i}\left(k\right)=\left\{\begin{array}{cc} \frac{{\hat{w}}_{i}\left(k+1\right)-{\hat{w}}_{i}\left(k\right)}{\Delta t}, & \mathrm{if}\;k=1\\ \frac{{\hat{w}}_{i}\left(k+1\right)-{\hat{w}}_{i}\left(k-1\right)}{2\Delta t}, & \mathrm{if}\;k=2\;\mathrm{or}\;k=T_{final}/\Delta t-1\\ \frac{{\hat{w}}_{i}\left(k\right)-{\hat{w}}_{i}\left(k-1\right)}{\Delta t}, & \mathrm{if}\;k=T_{final}/\Delta t\\ \frac{-{\hat{w}}_{i}\left(k+2\right)+8{\hat{w}}_{i}\left(k+1\right)-8{\hat{w}}_{i}\left(k-1\right)+{\hat{w}}_{i}\left(k-2\right)}{12\Delta t}, & \mathrm{otherwise} \end{array}\right..$$

In the final step, the projection and optical flow predictions, ${\left\{{\hat{w}}_{i}\left(k\right)\right\}}_{i=1}^{n_{f}}$ and ${\left\{{\hat{\dot{w}}}_{i}\left(k\right)\right\}}_{i=1}^{n_{f}}$, are utilized as the pseudo-measurements for UKF-GP during the long-term occlusion.

### 2.5. Simulation Workflow

The formal simulation workflow according to which we have verified the UKF-GP algorithm is provided in Algorithm 2. In the beginning, $n_{train}=T_{train}/\Delta t$ sensor measurements are collected from t=0 s till $t=T_{train}$ with the measurement frequency $f_{s}$, and we assume that occlusion happens from $t=T_{train}$ until the end of the simulation ($t=T_{total}$). The projection measurements obtained from t=0 until $t=T_{train}$ are stored in the datasets ${\left\{D_{i}\right\}}_{i=1}^{n_{f}}$. Next, the multi-output GP models ${\left\{G_{i}\right\}}_{i=1}^{n_{f}}$ are trained using the datasets, the product kernel, and the hyper-parameter initial guesses. Afterward, ${\left\{{\hat{w}}_{i}\right\}}_{i=1}^{n_{f}}$ are generated for the whole duration of the simulation, from t=0 s to $t=T_{total}$ ($T_{total}>T_{train}$) sec with the prediction sampling frequency $f_{GP}$. Next, ${\left\{{\hat{\dot{w}}}_{i}\left(k\right)\right\}}_{i=1}^{n_{f}}$ are calculated from ${\left\{{\hat{w}}_{i}\right\}}_{i=1}^{n_{f}}$ using FDM provided in Equation (27). Then, the UKF model is initiated with the discrete-time interval $\Delta t_{GP}=1/f_{GP}$, total simulation time $T_{total}$, the dynamics model f(X), the observation model h(X), the initial guesses of the state variable ${\hat{X}}_{0}$, the initial error covariance matrix $P_{0}$, the process noise covariance matrix Q, and the measurement noise covariance matrix R. Once the model is initiated, the state variables are updated using ${\left\{{\hat{w}}_{i}\left(k\right)\right\}}_{i=1}^{n_{f}}$ and ${\left\{{\hat{\dot{w}}}_{i}\left(k\right)\right\}}_{i=1}^{n_{f}}$ as the pseudo-measurements at every time-step until the end of the simulation.
**Algorithm 2** Simulation workflow.  1:From t=0 to $t=T_{train}$, collect $n_{train}$ number of sensor-provided feature projection measurements ${\left\{{\hat{w}}_{i}\left(k\right)\right\}}_{i=1}^{n_{f}}$ with the measurement frequency $f_{s}$.  2:Store the projection measurement data in the ${\left\{D_{i}\right\}}_{i=1}^{n_{f}}$ and the kernel functions.  3:Train $n_{f}$ number of multi-output GP models ${\left\{G_{i}\right\}}_{i=1}^{n_{f}}$ from data sets ${\left\{D_{i}\right\}}_{i=1}^{n_{f}}$.  4:From ${\left\{G_{i}\right\}}_{i=1}^{n_{f}}$, sample projection predictions ${\left\{{\hat{w}}_{R}={\left[{\hat{u}}_{R,i},{\hat{v}}_{R,i},{\hat{u}}_{L,i}\right]}^{T}\right\}}_{i=1}^{n_{f}}$ from t=0 to $t=T_{total}$ with the sampling frequency $f_{GP}$.  5:Calculate the optical flows ${\left\{{\hat{\dot{w}}}_{i}\left(k\right)\right\}}_{i=1}^{n_{f}}$ from ${\left\{{\hat{w}}_{i}\left(k\right)\right\}}_{i=1}^{n_{f}}$ according to Equation (27).  6:Initiate the UKF-GP model with $\Delta t_{GP}$, ${\hat{X}}_{0}$, $P_{0}$, Q, R, f(X), h(X), $T_{total}$.  7:**for** k=1 to $T_{total}/\Delta t_{GP}$ **do**  8:      Predict the state variables and the state error covariance matrix using the discrete dynamics model and the sigma points.  9:      Update the estimated state variables using the observation model with the GP-provided predicted projections ${\left\{{\hat{w}}_{i}\left(k\right)\right\}}_{i=1}^{n_{f}}$ and the optical flows ${\left\{{\hat{\dot{w}}}_{i}\left(k\right)\right\}}_{i=1}^{n_{f}}$.10:    k←k+111:**end for**

## 3. Results and Discussion

In order to demonstrate the effectiveness of the proposed UKF-GP algorithm over the conventional UKF model to estimate the rotational motion and inertia parameters of a non-cooperative and tumbling target space object, we conducted Monte Carlo (MC) simulations for different tumbling frequencies, and the results are presented in this section. First, we present the MC simulation results regarding the GP models to portray the prediction accuracy of the GP models, which are followed by the MC simulation results regarding the UKF-GP and conventional UKF algorithms.

The common simulation parameters for the MC simulations of GP and UKF-GP are provided below:

- Discrete-time intervals: $\Delta t=\Delta t_{gp}=1$ s;
- Sensor measurement sampling rate: $f_{s}=1$ Hz;
- Principal axes inertia parameters of the target: $I_{x}=90{\mathrm{kgm}}^{2}$, $I_{x}=100{\mathrm{kgm}}^{2}$, $I_{z}=112{\mathrm{kgm}}^{2}$;
- Tumbling frequencies: $f_{T}=0.025$, 0.075, 0.125, and 0.175 Hz (the corresponding polhode periods $T_{p}$ = 441.045 s, 147.015 s, 88.209 s, and 63.006 s, respectively);
- Initial angular velocities in B: ω0B=2πfT[0.67,−0.33,0.67]T rad/s;
- Initial attitude quaternion: $q_{0}={\left[1,0,0,0\right]}^{T}$;
- Standard deviation of the projection measurement: $\sigma_{p}=10^{-5}$ rad.

The sensor measurement frequency $f_{s}$ is assumed to be 1 Hz because visual camera sensors have a slower sampling frequency [1], and this value was considered by similar works that were previously conducted [6,7]. Both the tumbling frequency $f_{T}$ and the sensor measurement frequency $f_{s}$ influence the prediction performance of the GP models. We considered $f_{s}$ to be at least five times $f_{T}$, which we determined by trial and error using the following reasons. This criterion is set to ensure that the fluctuations of the projection measurements are properly captured in the training dataset and that the GP models can provide good predictions. Since $f_{s}=1$ Hz is assumed to be fixed in our work, $f_{T}$ should be below 0.20 Hz, and we considered the $f_{T}$ values in this study accordingly. Note that this is a drawback that is caused by the selected sensor with low measurement frequency, not by the GP algorithm itself. Additionally, the considered standard deviation of the projection measurements $\sigma_{p}$ is also assumed by previous similar studies [6,7]. Moreover, the necessary mathematical equations to calculate the polhode period $T_{p}$ given the principal inertia parameters and the initial angular velocity are provided in Appendix B and adapted from [34].

All results presented in this section were obtained by conducting simulations on a desktop computer with 12th Gen ${\mathrm{Intel}}^{®}{\mathrm{Core}}^{\mathrm{TM}}$ i7-12700 (3.60 GHz) and 32 GB of RAM. Additionally, we used a Python-based open-source Gaussian process framework, ‘GPy’ [36], for generating GP models. The hyper-parameter optimization was performed by implementing the ‘L-BFGS’ optimizer.

### 3.1. Prediction Performance of the GP

Before demonstrating the estimation performance of the proposed UKF-GP algorithm, we present the prediction accuracy of the GP models in varying scenarios. For this task, we conducted MC simulations for four tumbling frequencies and nine different training data time spans. The specific simulation parameters for these MC simulations are given below:

- Number of features: $n_{f}=1$;
- Position of the feature in B: pB=[1,1,1]T m;
- Training data time-span: $T_{train}=75$, 100, 200, 500, 1000, 1500, 2000, 2500, and 3000 s;
- Duration of prediction: $T_{predict}=T_{total}-T_{train}=2000$ s;
- Number of runs: $n_{run}=20$.

**Figure 4:**
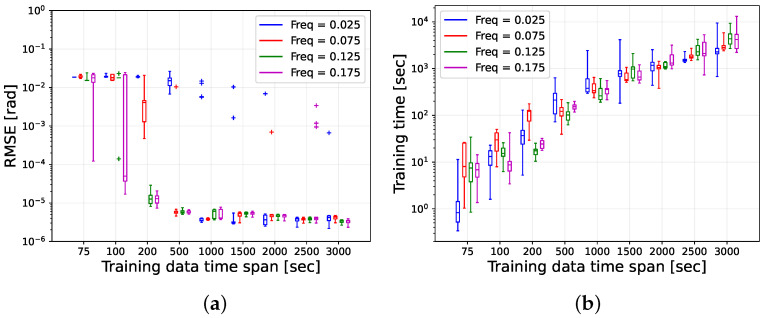
Results from the MC simulations for the GP models. (**a**) RMSE box plots of the predicted projections for 2000 s, and (**b**) the training time of the GP model.

To compare the prediction performances among the GP models in different scenarios, we calculated the root mean square error (RMSE) of the predicted projection vector ${\left\{{\hat{w}}_{i}\right\}}_{i=1}^{n_{f}}$ from $k=T_{train}/\Delta t$ to $k=(T_{train}+T_{predict})/\Delta t$, in other words, for 2000 time steps starting from $k=T_{train}/\Delta t$. The box plots of the RMSE distributions and the GP model train time for all MC runs are provided in Figure 4.

In Figure 4a, the RMSE vs. the training data time span $T_{train}$ box plots are provided, where we can observe the gradual decrease in the RMSEs for all $f_{T}$ as $T_{train}$ increases. For $f_{T}=0.025$ Hz and the first three values of $T_{train}$, significantly high RMSEs were observed, indicating that GP models are incapable of generating highly accurate projection predictions in these scenarios due to the lack of sufficient training data. However, for $T_{train}\geq 1000\;s\;\approx 2T_{p}$, GP models can predict projections with RMSEs on the scale of $10^{-6}$ rad, which shows the GP models’ prediction accuracy with longer $T_{train}$.

For $f_{T}=0.075$ Hz, a similar trend of the RMSE over $T_{train}$ was observed, but the drop of the RMSEs occured at a smaller value of $T_{train}$. For $T_{train}=75$ and 100 s, the RMSEs were very high but suddenly dropped below $10^{-5}$ rad at $T_{train}=200\;s\;\approx 3.4T_{p}$. For higher $T_{train}$, the RMSEs remained below $10^{-5}$ rad, which illustrated the highly accurate prediction ability of the GP models in these scenarios.

For $f_{T}=0.125$ and $T_{train}=75$ and 100 s, the RMSEs were quite high due to insufficient training data. However, starting from $T_{train}=200\;s\;\approx 2.26T_{p}$, the RMSE distribution was mostly on the scale of $10^{-5}$ rad and below $10^{-5}$ rad for higher $T_{train}$.

The box plots for $f_{T}=0.175$ Hz at smaller values of $T_{train}$ show a more interesting trend. While for all other $f_{T}$ at $T_{train}=75$ and 100 s, the spans of the RMSE box plots were very narrow, the spans for $f_{T}=0.175$ Hz at these $T_{train}$ were quite long. These long spans of the box plots indicate that even at these smaller $T_{train}$ and smaller-sized datasets, the GP models in some of the MC runs managed to be trained properly; therefore, in those runs, the prediction accuracy was acceptable. In particular, at $T_{train}=100$ s, in more than 50% of the runs, the GP models generated good projection predictions as the median line was observed to be in the scale $10^{-5}$ rad. For $T_{train}=200$ s $\approx 3.17T_{p}$, the RMSE distribution was mostly on the scale of $10^{-5}$ rad, which was below $10^{-5}$ for $T_{train}\geq 500$ s.

From the above analysis, it is evident that the minimum duration of the training data time span $T_{train}$ to create GP models capable of generating quality predicted projections is about $3T_{p}$. However, this empirical relationship between $T_{train}$ and $T_{p}$ is only applicable to the considered $f_{T}$. A general expression for the minimum $T_{train}$ to create capable GP models requires a more exhaustive investigation.

In Figure 4a, several anomalies can be observed for all tumbling frequencies except $f_{T}=0.125$ Hz. These anomalous GP models were not properly trained because, occasionally, the optimization algorithm got stuck at some local minima during the hyper-parameter training process; thus, non-optimal values were selected as the model hyper-parameters. The occurrence of these anomalies may have been avoided if a global optimizer was used, which is a more computationally expensive choice.

The training time box plots provided in Figure 4b exhibit that the training time spanning from a few seconds to a few hours has a rather logarithmic relationship with the training data time span regardless of the choice of $f_{T}$. In combination, Figure 4a,b helps to conclude that $T_{train}=1000$ s is the optimal choice of $T_{train}$ to train good GP models, which are capable of providing highly accurate predictions for thousands of seconds while the model training time requires only a few minutes.

### 3.2. Prediction Performance of UKF-GP

The MC simulations to demonstrate the effectiveness of the UKF-GP algorithm were conducted for four $f_{T}$ values and one $T_{train}$ value. The details of the MC simulations of the UKF-GP models are provided below:

- Total simulation duration: $T_{total}=3000$ s.
- Duration of the sensor data availability: $T_{train}=1500$ s.
- Duration of the occlusion: $T_{predict}=1500$ s.
- Number of features: $n_{f}=5$.
- User-defined constant parameters: α=0.1, β=2, κ=0 (from [26]).
- Position of the features in B:
  – Feature 1: p1B=[0.1302,−0.6648,−0.2264]T m;
  – Feature 2: p2B=[1.0343,−1.4858,−1.1352]T m;
  – Feature 3: p3B=[0.5122,0.9775,−1.0898]T m;
  – Feature 4: p4B=[0.2252,1.1739,−0.8723]T m;
  – Feature 5: p5B=[−0.9440,−1.1748,−0.8409]T m.
- Initial guesses of the state variables:
  – ω^0B=1.75ωB(0) rad/s;
  – ${\hat{q}}_{0}={\left[0.9707,0.1386,0.13860,0.1386\right]}^{T}$;
  – ${\hat{k}}_{1}={\hat{k}}_{2}=0$;
  – p^iB=piB+[0.7,0.7,0.7]T m.
- Standard deviation of the measurement noise of the optical flow:
  – For $f_{T}=0.025$ Hz, $\sigma_{w}=10^{-5}$ rad/s;
  – For $f_{T}=0.075$ Hz, $\sigma_{w}=5\times 10^{-4}$ rad/s;
  – For $f_{T}=0.125$ Hz, $\sigma_{w}=5\times 10^{-4}$ rad/s;
  – For $f_{T}=0.175$ Hz, $\sigma_{w}=10^{-3}$ rad/s.
- Standard deviation of the measurement noise of the disparity: $\sigma_{d}=\sigma_{p}=10^{-5}$ rad.
- Variances for the initial state error covariance matrix:
  – Variance of ωB: $\Sigma_{\omega }^{P}=0.1225\left[1,1,1\right]{\mathrm{rad}}^{2}{\mathrm{/s}}^{2}$;
  – Variance of q: $\Sigma_{q}^{P}=0.04\left[1,1,1,1\right]$;
  – Variance of $k_{1}$ and $k_{2}$: $\Sigma_{k_{1}}^{P}=\Sigma_{k_{2}}^{P}=0.01$;
  – Variance of piB: $\Sigma_{p_{i}}^{P}=2.25\left[1,1,1\right]m^{2}$.
- Variances in the process noise covariance matrix:
  – Variance in ωB:
    ∗ For $f_{T}=0.025$ Hz, $\Sigma_{\omega }^{Q}=2.5\times 10^{-9}\left[1,1,1\right]{\mathrm{rad}}^{2}{\mathrm{/s}}^{2}$;
    ∗ For $f_{T}=0.075$ Hz, $\Sigma_{\omega }^{Q}=2.5\times 10^{-7}\left[1,1,1\right]{\mathrm{rad}}^{2}{\mathrm{/s}}^{2}$;
    ∗ For $f_{T}=0.125$ Hz, $\Sigma_{\omega }^{Q}=2.5\times 10^{-5}\left[1,1,1\right]{\mathrm{rad}}^{2}{\mathrm{/s}}^{2}$;
    ∗ For $f_{T}=0.175$ Hz, $\Sigma_{\omega }^{Q}=2.5\times 10^{-5}\left[1,1,1\right]{\mathrm{rad}}^{2}{\mathrm{/s}}^{2}$.
  – Variance in q:
    ∗ For $f_{T}=0.025$ Hz, $\Sigma_{q}^{Q}=4\times 10^{-10}\left[1,1,1,1\right]$;
    ∗ For $f_{T}=0.075$ Hz, $\Sigma_{q}^{Q}=4\times 10^{-8}\left[1,1,1,1\right]$;
    ∗ For $f_{T}=0.125$ Hz, $\Sigma_{q}^{Q}=4\times 10^{-6}\left[1,1,1,1\right]$;
    ∗ For $f_{T}=0.175$ Hz, $\Sigma_{q}^{Q}=4\times 10^{-6}\left[1,1,1,1\right]$.
  – Variance in $k_{1}$ and $k_{2}$: $\Sigma_{k_{1}}^{Q}=\Sigma_{k_{2}}^{Q}=3.6\times 10^{-5}$.
  – Variance in piB: $\Sigma_{p_{i}}^{Q}=10^{-6}\left[1,1,1\right]m^{2}$.

In this study, the selection of the number of features $n_{f}$ was adopted from a previous study [7]. Also, the standard deviations of the optical flow measurement noise were obtained from FDM. Additionally, the diagonal variance components of $P_{0}$ and Q matrices were chosen manually by trial and error.

To illustrate the superiority of the UKF-GP model over the conventional UKF model, we conducted MC simulations of UKF under long-term occlusion with the same setup as that of UKF-GP. Moreover, MC simulations of the UKF model without long-term occlusion were also performed, and the results are presented along with UKF and UKF-GP as a benchmark so that the reader can easily compare the superior performance of UKF-GP to that of UKF. In the subsequent figures, the results of UKF with and without long-term occlusion are denoted by the ’UKF’ and ’Benchmark’, respectively.

**Figure 5:**
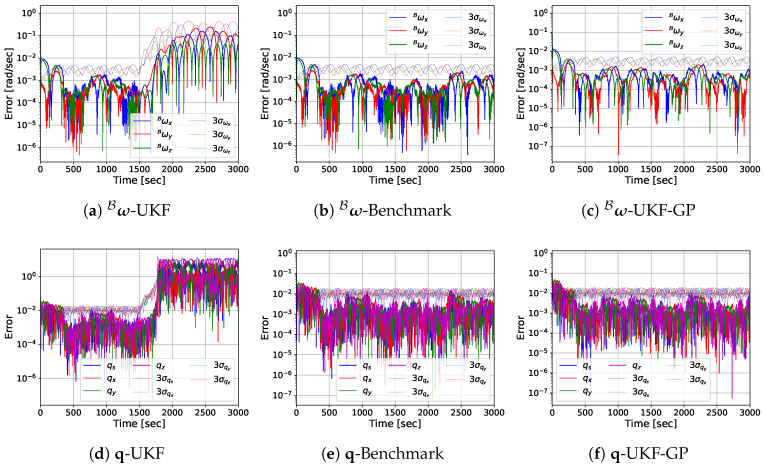

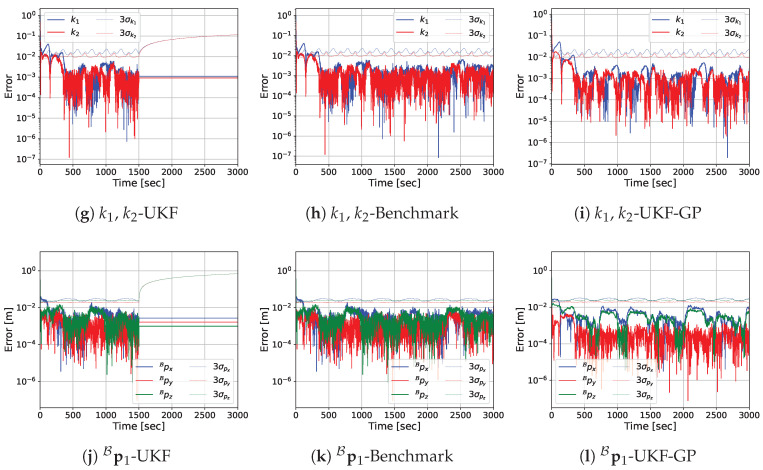
State variable estimation errors of UKF, Benchmark, and UKF-GP for $f_{T}=0.025$ Hz.

**Figure 6:**
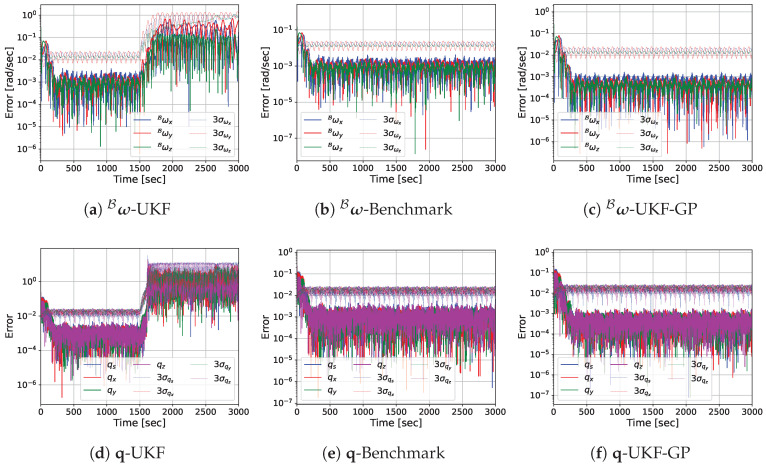

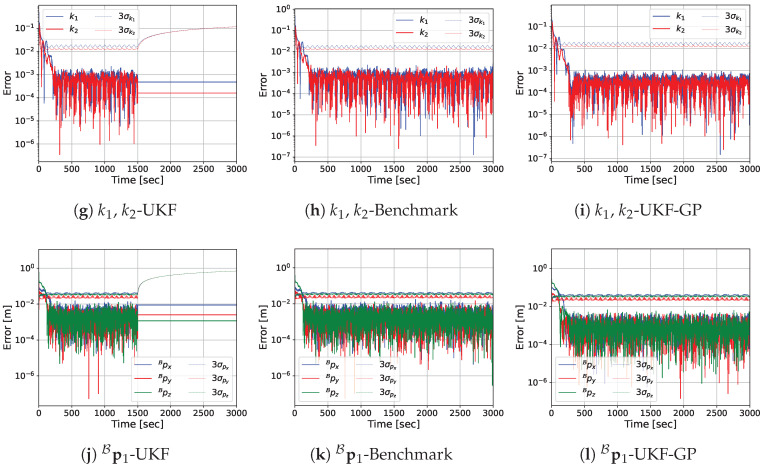
State variable estimation errors of UKF, Benchmark, and UKF-GP for $f_{T}=0.075$ Hz.

**Figure 7:**
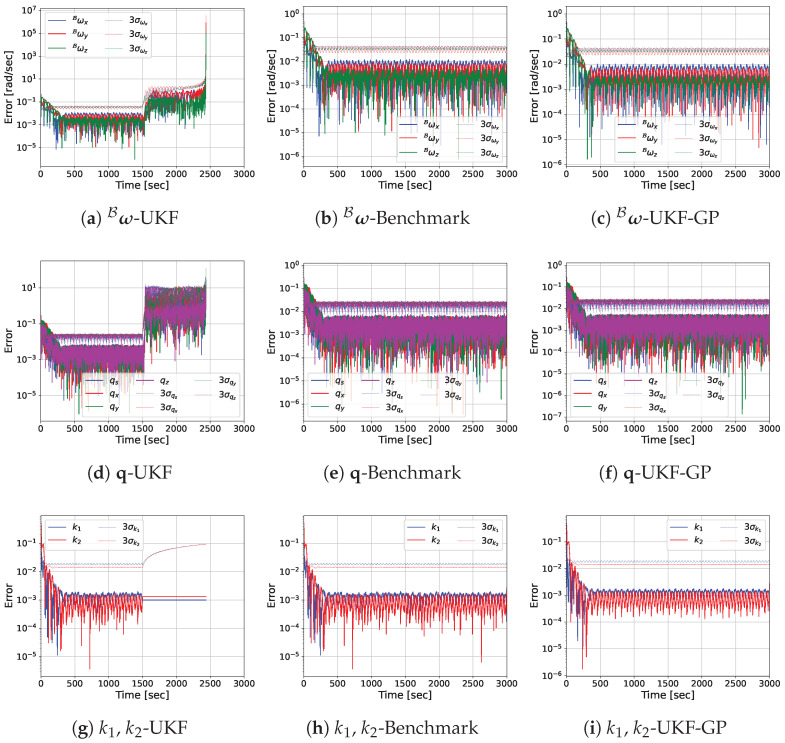

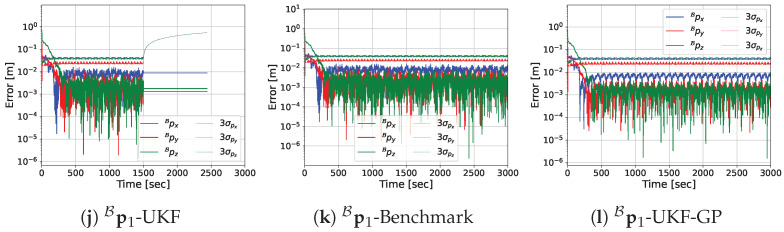
State variable estimation errors of UKF, Benchmark, and UKF-GP for $f_{T}=0.125$ Hz.

**Figure 8:**
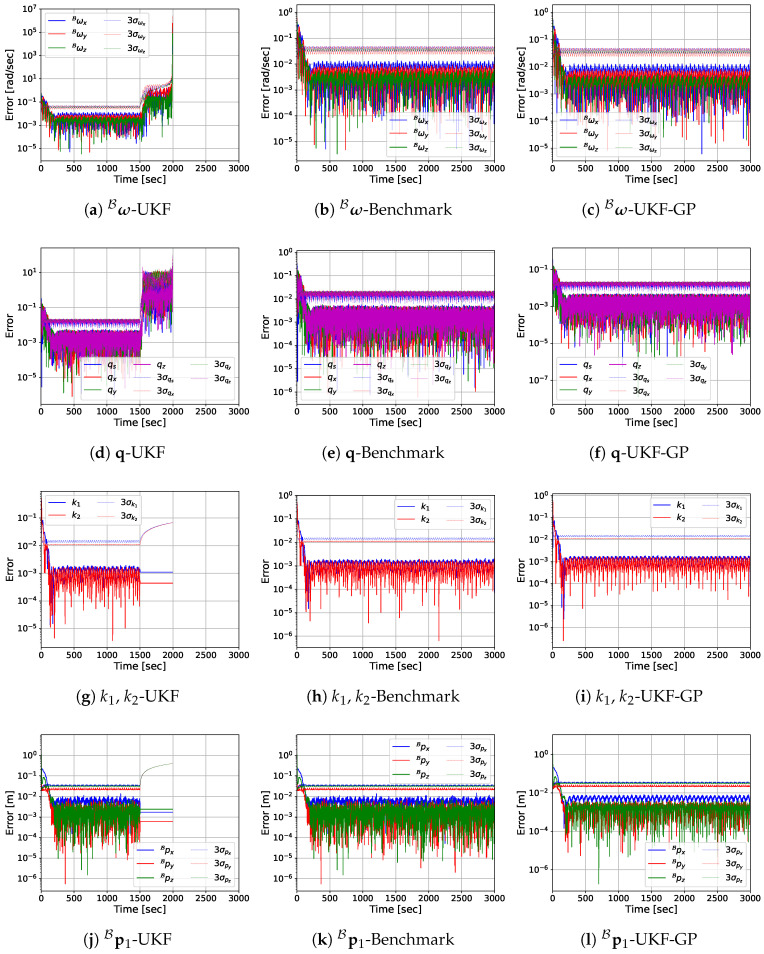
State variable estimation errors of UKF, Benchmark, and UKF-GP for $f_{T}=0.175$ Hz.

Before presenting the MC simulation results, we provide the estimation error performances of UKF, Benchmark, and UKF-GP for a single run so that the reader can easily observe the performance differences among the considered approaches. The log-scale plots of the absolute estimation errors of ω^B, $\hat{q}$, ${\hat{k}}_{1}$, ${\hat{k}}_{2}$, and p^1B obtained from UKF, Benchmark, and UKF-GP are provided in Figure 5, Figure 6, Figure 7 and Figure 8 for $f_{T}=0.025$, 0.075, 0.125, and 0.175 Hz, respectively.

Figure 5 shows that for $f_{T}=0.025$ Hz and UKF, the absolute estimation errors of the ω^B components rapidly decrease at the beginning and mostly stay below $10^{-3}$ rad/s from t=300 s until the beginning of occlusion at t=1500 s, which is the same for Benchmark. However, once the occlusion starts at t=1500 s, the estimation errors rapidly diverge, and the upper limit of the errors reaches the scale of $10^{-1}$ rad/s. The divergence of error occurs because the UKF model cannot be updated during occlusion due to the lack of stereo-camera observations; hence, only the propagation of the ω^B according to the dynamics model cannot provide reliable estimates. Similar behavior was observed for the estimation error of $\hat{q}$, where the errors quickly converge at the beginning and the errors remain below $10^{-2}$, but during occlusion, the diverging errors exceed the value of 1. The state variables $k_{1}$, $k_{2}$, and p1B are constant parameters which do not change with time. Hence, their estimates ${\hat{k}}_{1}$, ${\hat{k}}_{2}$, and p^1B keep converging before occlusion because of the availability of the camera measurements, but during occlusion, they retain their values after the last update. Since the estimation errors of these parameters already reach smaller values ($\approx 10^{-3}$) by the time occlusion is initiated, they remain the same throughout the entire span of occlusion.

From Figure 5, we observe that the performance of UKF-GP is very similar to that of UKF and Benchmark before occlusion, as the estimation errors of the state variables rapidly converge at the beginning and remain small before occlusion. However, the main contrast between UKF-GP and UKF is clearly visible during occlusion. Unlike UKF, UKF-GP manages to stop the divergence of the rotational motion variable estimates ω^B and $\hat{q}$ during occlusion and the errors are comparable with that of Benchmark. Also, the errors are confined by the 3−σ uncertainty boundaries for UKF-GP during occlusion, which is not observed for UKF. These improvements result from the highly accurate projection estimates ${\left\{{\hat{w}}_{i}\right\}}_{i=1}^{n_{f}}$ from the GP models and the optical flow estimates ${\left\{{\hat{\dot{w}}}_{i}\right\}}_{i=1}^{n_{f}}$, which are used as the pseudo-measurements for UKF-GP during occlusion. For ${\hat{k}}_{1}$, ${\hat{k}}_{2}$, and p^1B, the estimation errors of UKF-GP also mimic that of Benchmark.

For $f_{T}=0.075$ Hz, the state estimation errors from UKF and UKF-GP behave similarly to the previous scenario, as seen in Figure 6. The estimation errors of ω^B and $\hat{q}$ from UKF converge at the beginning and remain small before t=1500 s for UKF but quickly diverge to considerably high values during occlusion. Contrary to that, the state estimation errors of UKF-GP remain significantly small throughout the entire duration except for the initial convergence, which is similar to Benchmark.

The most notable observation from the estimation errors plots for $f_{T}=0.125$ Hz in Figure 7 is that ω^B errors diverge exponentially from t=2000 s to t=2438 s for UKF during occlusion. At the end, the order of magnitude of ω^B errors reaches 6. The simulation is forced to stop here because the covariances of ω^B reaches virtually infinity at this point. During occlusion, the errors of $\hat{q}$ for UKF also become significantly high, whereas the other state variable errors remain constant and small. On the other hand, UKF-GP continues to provide reliable estimates of all state variables as before throughout the entire simulation. Similar observations about the state estimations errors from UKF and UKF-GP are made for $f_{T}=0.175$ Hz, except the UKF simulation stops at t=1993 s due to the extremely high covariances associated with ω^B.

**Figure 9:**
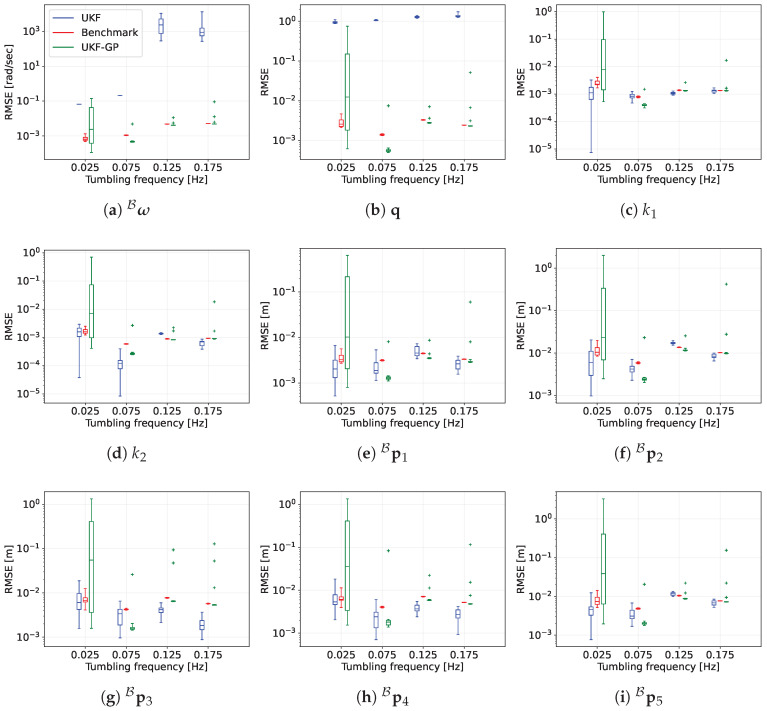
RMSEs of the state estimation errors of UKF, Benchmark, and UKF-GP for 1500 s of occlusion from MC simulations. All figures have the same legend; therefore, the legend is only provided in (**a**).

The MC simulation results of UKF, Benchmark, and UKF-GP for four $f_{T}$ values are provided in Figure 9, where we present the box plots of the RMSEs of the estimations of the state variables during occlusion from t=1500 s until t=3000 s. First, we will point our focus towards the box plots corresponding to the RMSEs of ω^B shown in Figure 9a. The RMSE distributions of UKF are located at the higher orders of magnitudes (≈−1 for 0.025 Hz and 0.075 Hz and 4 to 5 for 0.125 Hz and 0.175 Hz) compared to the RMSE distributions of Benchmark. This discrepancy indicates that the UKF-estimated ω^B diverges during occlusion for all runs and all tumbling frequencies. The significantly high magnitudes of the RMSE of UKF at the higher tumbling frequencies also indicate that at the end of the simulations, ω^B diverges exponentially, which we have observed in Figure 7 and Figure 8. However, for UKF-GP, the RMSE distributions are either below (0.075 Hz) or at the same level as the distributions of Benchmark (0.125 Hz and 0.175 Hz) at all $f_{T}$ values except 0.025 Hz.

The RSME distribution of UKF-GP at 0.025 Hz spans from the scale of $10^{-4}$ rad/s to $10^{-1}$ rad/s, and the median is on the scale of $10^{-3}$ rad/s. This long span is due to the fact that among 20 runs, UKF-GP provides reliable ω^B in 10 runs, and in the other 10 runs, UKF-GP is not so accurate at the estimation task. It turns out that among a total of 100 GP models (five GP models for five features per run), 15 GP models in 10 runs could not be trained properly, which affects the estimation performance of UKF-GP in those runs. UKF-GP is largely affected by this issue, particularly at $f_{T}=0.025$ Hz because the corresponding $T_{p}=441.045$ s, which means that $T_{train}=1500$ s is only 3.4 times $T_{p}$, whereas for the other frequencies with shorter $T_{p}$, $T_{train}$ is 10.2, 17.005, 23.807 times $T_{p}$. With a relatively small number of training data, the GP models get less chance to learn about the underlying periodic trends from the training data for $f_{T}=0.025$ Hz compared to the other $f_{T}$ values.

Similar trends are observed for the RMSE box plots of $\hat{q}$ in Figure 9b. The RMSEs of UKF in all runs have significantly higher values (on a scale of 1) compared to that of Benchmark (on a scale of $10^{-3}$). Except for 0.025 Hz, UKF-GP’s RMSE boxes are either significantly below (0.075 Hz) or close to the boxes of Benchmark (0.125 Hz and 0.175 Hz). The box of UKF-GP at 0.025 Hz has a very long span due to some improperly trained GP models, as we have explained above.

The box plots for ${\hat{k}}_{1}$ and ${\hat{k}}_{2}$ in Figure 9c,d show that the RMSEs of UKF are in smaller scales ($10^{-3}$ or less), unlike the rotational motion variables. This happens for two reasons: first, the $k_{1}$ and $k_{2}$ are inertia parameters that do not change with time and during occlusion, their estimations cannot be updated anymore, resulting in constant errors. Second, by the time when the occlusion starts (t=1500 s), the ${\hat{k}}_{1}$ and ${\hat{k}}_{2}$ have already reached close to the truth, and the errors have become quite small. Therefore, the errors remain constant and small during the occlusion, and as a result, the order of magnitude of the corresponding RMSEs is small. The RMSEs of UKF-GP also remain small and are comparable to Benchmark for all tumbling frequencies except for 0.025 Hz.

Finally, the box plots for {p^iB}i=15 are provided in Figure 9e–i. For UKF, the errors do not change during the occlusion because of the reason explained above, and as a result, the corresponding RMSEs are quite small ($10^{-2}$ m or smaller). Similarly, the RMSEs of UKF-GP also remain small ($10^{-2}$ m or smaller) except 0.025 Hz.

For UKF-GP and all $f_{T}$ except 0.025 Hz, we can observe a small number of anomalies, which are indicated by the ‘+’ symbol in Figure 9. These anomalies are attributed to a few GP models that are not trained properly. These GP models could not provide highly accurate estimates of the projections of the corresponding features, which affected the estimation accuracy of UKF-GP.

## 4. Conclusions

This study proposes an unscented Kalman filter–Gaussian process (UKF-GP) fusion algorithm, which provides reliable estimates of the rotational motion variables and the inertia parameters of a non-cooperative tumbling target space object under long-term occlusions (unavailability of sensor measurements for hundreds or thousands of seconds). We assumed that a stereo-camera system onboard a chaser spacecraft provides projection information of multiple point features of the target. The collected projection data were used as training data for multi-output GP models, and we utilized periodic kernels to capture the periodic trends from non-periodic and noisy projection measurements. The initial guesses of the periodicity hyper-parameters were obtained from the fast Fourier transform (FFT) analysis of the projection data as a way to improve the hyper-parameter training procedure. Once trained, the GP models provided the predicted projection information regarding the features. Then, optical flows were obtained from the finite difference method (FDM) and predicted projections. Finally, predicted projections and optical flows were used as the pseudo-measurements for the UKF-GP algorithm during long-term occlusion.

To validate the capabilities of the GP models and the proposed UKF-GP algorithm, Monte Carlo (MC) simulations were performed for four tumbling frequencies: 0.025, 0.075, 0.125, and 0.175 Hz. The MC simulations of the GP models demonstrated that the GP models can provide highly accurate projection prediction (RMSEs in the scale of $10^{-6}$ rad) for 2000 s and for all considered tumbling frequencies if the time span of the training data is approximately three times the polhode period or longer. The MC simulations conducted for UKF-GP show that throughout the entire duration of the simulations and for all tumbling frequencies except 0.025 Hz, UKF-GP can provide reliable estimates of the rotational motion variables for 1500 s long occlusion, which are as good as the estimates from Benchmark (conventional UKF without the long term occlusion). On the contrary, the estimates from the conventional UKF algorithm diverged once occlusion begins. It was also observed that both the UKF and UKF-GP algorithms can provide good estimates of the inertia parameters and the feature positions in the body frame during occlusion, except for UKF-GP at 0.025 Hz. UKF-GP cannot perform well in 50% of the runs for the tumbling frequency of 0.025 Hz because of the lack of sufficient training data. Overall, it is evident from the results that the UKF-GP algorithm outperforms the conventional UKF algorithm by a large margin in estimating the rotational motion variables and the inertia parameters of a non-cooperative tumbling target object under long-term occlusions.

In the future, we plan to enhance the UKF-GP algorithm so that both the relative rotational and translational motion variables of a target space object can be estimated during long-term occlusions. Also, we aim to make the UKF-GP algorithm compatible with a varying number of features instead of a fixed number of features, as assumed in this study. Finally, we will validate the effectiveness of the UKF-GP algorithm in an experimental setup.

## Figures and Tables

**Table 1:** Format of the training dataset $D_{i}$.

Input	Output		
Time, * t *	Projection Components, ${\tilde{w}}_{i}$ [rad]		
[sec]	$u_{R}$ Component	$v_{R}$ Component	$u_{L}$ Component
t(1)	${\tilde{u}}_{R,i}\left(1\right)$	${\tilde{v}}_{R,i}\left(1\right)$	${\tilde{u}}_{L,i}\left(1\right)$
t(2)	${\tilde{u}}_{R,i}\left(2\right)$	${\tilde{v}}_{R,i}\left(2\right)$	${\tilde{u}}_{L,i}\left(2\right)$
⋮	⋮	⋮	⋮
t(k)	${\tilde{u}}_{R,i}\left(k\right)$	${\tilde{v}}_{R,i}\left(k\right)$	${\tilde{u}}_{L,i}\left(k\right)$

## Data Availability

The datasets presented in this article are not readily available because the data are part of an ongoing study. Requests to access the datasets should be directed to xiaoli.bai@rutgers.edu.

## Abbreviations

The following abbreviations are used in this manuscript:

UKF	Unscented Kalman filter
GP	Gaussian process
FFT	Fast Fourier transform
LCS	Laser camera system
EKF	Extended Kalman filter
IEKF	Iterated extended Kalman filter
MC	Monte Carlo
CoMBiNa	Coarse model-based relative navigation
SLAM	Simultaneous localization and mapping
ML	Machine learning
SPGP	Sparse pseudo-input Gaussian process
RBF	Radial basis function
DVQ-MANN	Dual-vector quaternion-based mixed artificial neural network
DVQ-EKF	Dual-vector quaternion-based extended Kalman filter
CM	Center of mass
COP	Center of projection
SURF	Sped-up robust features
SIFT	Scale invariant feature transform
L-BFGS	Limited-memory Broyden–Fletcher–Goldfarb–Shanno
RMSE	Root mean square error

## Appendix A. Determination of Expressions of ${\dot{w}}_{R}$ and ${\dot{w}}_{L}$

For the projections on the right camera plane, we first take the time derivatives of $u_{R,i}$ and $v_{R,i}$:

(A1) $${\dot{u}}_{R,i}=f\left[\frac{\dot{x_{i}}}{y_{i}}-\frac{x_{i}}{y_{i}^{2}}\dot{y_{i}}\right],$$

(A2) $${\dot{v}}_{R,i}=f\left[\frac{\dot{z_{i}}}{y_{i}}-\frac{z_{i}}{y_{i}^{2}}\dot{y_{i}}\right].$$

Now, $\dot{x_{i}}$, $\dot{y_{i}}$, and $\dot{z_{i}}$ are the components of the rate of change in ρiI, which is the position of the feature w. r. t., the chaser CM expressed in I. The expression for ρiI is repeated below:

(A3) ρiI=ρ0I+[CIB]T·piB=ρ0I+piI.

Since ρ0I is constant, ρ˙iI is zero. Therefore, we can write the velocity expression as follows:

(A4) vI=ρ˙iI=ωI×piI=ωI×(ρiI−ρiI),

(A5) ⇒x˙iy˙iz˙i=ωyI(zi−z0)−ωzI(yi−y0)ωzI(xi−x0)−ωxI(zi−z0)ωxI(yi−y0)−ωyI(xi−x0).

Replacing ${\dot{x}}_{i}$ and ${\dot{y}}_{i}$ in Equation (A1), we get

(A6) u˙R,i=fxi˙yi−xiyi2yi˙=fωyI(zi−z0)−Iωz(yi−y0)yi−xiyi2(Iωz(xi−x0)−Iωx(zi−z0))⇒u˙R,i=fxi(zi−z0)yi2ωxI+(zi−z0)yiωyI−yi−y0yi+xi(xi−x0)yi2ωzI.

Similarly, for ${\dot{v}}_{R}$, we can write the following:

v˙R,i=fωxI(yi−y0)−Iωy(xi−x0)yi−ziyi2(Iωz(xi−x0)−Iωx(zi−z0)))=fyi−y0yi+zi(zi−z0)yi2ωxI−(xi−x0)yiωyI−zi(xi−x0)yi2ωzI.

Together, ${\dot{u}}_{R,i}$ and ${\dot{v}}_{R,i}$ can be written in the matrix format as follows:

(A7) w˙R,i=u˙R,iv˙R,i=AR,i·ωI=xi(zi−z0)yi2(zi−z0)yi−yi−y0yi+xi(xi−x0)yi2yi−y0yi+zi(zi−z0)yi2−(xi−x0)yi−zi(xi−x0)yi2ωxIωyIωzI.

The expression for ${\dot{w}}_{L,i}$ is very similar to that of ${\dot{w}}_{R,i}$ with a slight change to account for the distance between the cameras. The expression can be obtained simply by replacing $x_{0}$ and $x_{i}$ with $x_{0}+b$ and $x_{i}+b$ in Equation (A7). Note that ${\dot{v}}_{L,i}={\dot{v}}_{R,i}$ since $v_{L,i}=v_{R,i}$.

(A8) w˙L,i=u˙Lv˙L=AL,i·ωI=(xi+b)(zi−z0)yi2(zi−z0)yi−yi−y0yi+(xi+b)(xi−x0)yi2yi−y0yi+zi(zi−z0)yi2−(xi−x0)yi−zi(xi−x0)yi2ωxIωyIωzI.

## Appendix B. Determination of the Polhode Period $T_{p}$

Given that an object with the principal inertia parameters $I_{1}$, $I_{2}$, and $I_{3}$ is experiencing a torque-free tumbling motion, the magnitude of the angular momentum M and the total rotational energy *E* can be calculated from the following equations:

(A9) M=||M||2=||I·ω0B||2=I12ωx,0B+I22ωy,0B+I32ωz,0B,

(A10) E=12ω0B·I·ω0B=12(I12ωx,02B+I22ωy,02B+I32ωz,02B).

Then, considering that $I_{1}<I_{2}<I_{3}$ and $M^{2}>2EI_{2}$, the polhode period of ωB is given as follows:

(A11) $$T\left(E,M,I_{1},I_{2},I_{3}\right)=\frac{4K(m{\left(E,M,I_{1},I_{2},I_{3}\right)}^{2})}{\sqrt{\frac{\left(I_{3}-I_{2}\right)\left(M^{2}-2EI_{1}\right)}{I_{1}I_{2}I_{3}}}},$$

where

(A12) $$m\left(E,M,I_{1},I_{2},I_{3}\right)=\sqrt{\frac{\left(I_{2}-I_{1}\right)\left(2EI_{3}-M^{2}\right)}{\left(I_{3}-I_{2}\right)\left(M^{2}-2EI_{1}\right)}},$$

and $K(m^{2})$ is the Complete Elliptic Integral of the First Kind which is mathematically written as

(A13) $$K\left(m^{2}\right)=\int_{0}^{\pi /2}\frac{d\theta }{\sqrt{1-m^{2}\sin^{2}\theta }}.$$
